# A new approach in the treatment of ultrasound-guided synovial hypertrophy

**DOI:** 10.55730/1300-0144.5955

**Published:** 2024-11-11

**Authors:** Emel GÜLER, Alper DOĞANCI

**Affiliations:** 1 Division of Pain Management, Department of Physical Therapy and Rehabilitation, Sivas Cumhuriyet University, Sivas, Turkiye; 2 Division of Rheumatology, Department of Physical Therapy and Rehabilitation, Faculty of Medicine, Sivas Cumhuriyet University Sivas, Turkiye

**Keywords:** Knee osteoarthritis, synovial hypertrophy, ultrasound, injection

## Abstract

**Background/aim:**

Knee osteoarthritis (OA) causes pain and limited movement, negatively impacting daily life in older adults. Hypertrophy and changes in the synovial tissue significantly contribute to the pain. While intraarticular injections are common in OA treatment, specific therapies for hypertrophic tissue are rarely mentioned. This study aimed to evaluate the long-term outcomes of local anesthetic and steroid injections in the knee’s intraarticular space and hypertrophic synovial tissue.

**Materials and methods:**

Our retrospective study included patients with grade 3 or 4 knee OA diagnosed with ultrasound-guided suprapatellar effusion and synovial hypertrophy. Pain was assessed using a numerical rating pain scale (NRS) and functional capacity was evaluated with the Western Ontario and McMaster Universities Osteoarthritis (WOMAC) Index. Effusion was first drained from the suprapatellar region using a 22-gauge spinal needle under ultrasound guidance, followed by lavage with 40 mL of 0.9% NaCl solution. A mixture of 10 mL (2 mL triamcinolone hexacetonide, 7 mL of prilocaine, and 1 mL of 0.9% NaCl) was injected intraarticularly, and 6 mL was injected into the hypertrophic synovial tissue. Patients were followed before the injection and at 1, 3, 6, 9, and 12 months after the injection.

**Results:**

Analysis of the WOMAC scores and NRS values at 1, 3, 6, 9, and 12 months after the injection revealed statistically significant reductions (p < 0.05). No statistical difference was found between the duration of complaints and WOMAC scores or NRS values (p > 0.05). Ultrasound evaluation indicated regression of the synovial hypertrophy tissue.

**Conclusion:**

This injection method, practiced in the treatment of synovial hypertrophy as one of the causes of pain in knee OA, reduced pain and significantly increased functional capacity.

## 1. Introduction

Osteoarthritis (OA), the most common form of arthritis, is a leading cause of disability in older adults. The disease mechanisms include focal cartilage loss on the articular surface, osteophyte formation, subchondral bone remodeling, and thickening of the synovium and joint capsule. The hip, knee, spine, and hand joints are most commonly affected, with knee OA accounting for approximately 80% of cases [1]. The global prevalence of knee OA is about 16% [2]. Synovial hypertrophy in OA-affected joints plays a crucial role in the disease’s pathogenesis [3,4]. Histopathological features in the synovium include stromal vascularization, synovial inflammation, and hypertrophy resulting from mechanical stress and chronic low-grade inflammation. As a result, pain is observed [5]. Studies have shown that treatments involving steroids, stem cells, and immunosuppressive agents can suppress inflammation, reduce pain, and affect synovial tissue volume [6,7]. However, there is a limited body of literature specifically addressing the treatment of synovial hypertrophy.

Local anesthetics are commonly used in intraarticular arthritis treatment to block sensory nerves in the joint and surrounding anatomical structures, thereby reducing pain [8]. They are particularly effective in managing acute pain in the postoperative period as part of regional anesthesia techniques [9]. In clinical practice, the use of local anesthetics during knee intraarticular injections is widespread.

Synovial inflammation and resulting synovial hypertrophy in knee OA are painful conditions that negatively affect functional capacity. The optimal treatment approach for synovial hypertrophy and the choice of therapeutic agents remain unclear. This study aimed to demonstrate the outcomes of using a local anesthetic and steroid mixture in patients with knee OA with suprapatellar fluid increase and synovial hypertrophy. These results are based on 12-month follow-up periods with assessments of functional capacity, pain, and changes in synovial tissue.

## 2. Materials and methods

To conduct our retrospective study, we first applied to the University Hospital’s Physical Therapy and Rehabilitation Department for permission between June 1, 2021, and June 30, 2022. The study was approved by the local ethics committee (Date: 27.07.2022, Number: 20.07.2022) and performed in line with the principles of the Declaration of Helsinki. Patients diagnosed with grade 3 or 4 knee osteoarthritis according to Kellgren–Lawrence staging via direct X-ray as confirmed by ultrasound examination for suprapatellar effusion and synovial hypertrophy were included. The inclusion criteria were as follows: aged between 55 and 74 years, diagnosed with knee OA, and exhibiting suprapatellar effusion and synovial hypertrophy on ultrasound evaluation. Exclusion criteria included patients who did not consent to the use of their recorded data or had insufficient mental capacity to consent. Patients with a history of knee surgery or a history of previous infection and those diagnosed with inflammatory arthritis (rheumatoid arthritis, crystal arthropathy, gout, etc.) were excluded from the study. Patient data such as height, weight, age, and sex were recorded. Body mass index was defined as weight (kg)/height squared (m^2^). The procedure began with the patient in a supine position, with the knee flexed at an angle of 5° to 10° prior to the injection. Ultrasound imaging was then conducted on the lateral part of the knee using a sonography device (Voluson Ultrasound, GE Healthcare, Chicago, IL, USA) with a linear array probe operating at a frequency of 5–12 MHz. This sonographic procedure was performed by a physician with over 10 years of experience in musculoskeletal ultrasound in the rehabilitation medicine department.

In the treatment protocol applied for the patients, effusion was drained from the suprapatellar region using a needle under ultrasound guidance. This area was then aspirated and lavaged with 40 mL of 0.9% NaCl solution. Subsequently, 4 mL of a mixture totaling 10 mL, which consisted of 2 mL of triamcinolone hexacetonide (40 mg/mL steroid), 7 mL of prilocaine (20 mg/mL local anesthetic), and 1 mL of 0.9% NaCl, was injected into the intraarticular space, and 6 mL of the same mixture was injected into the hypertrophic synovial tissue (Figure). Following the performed procedures, no adverse effects related to the medication or the intervention were observed in the patients during follow-up. Patients were informed that they could use acetaminophen if needed.

### 2.1. Rating scales

#### 2.1.1. Kellgren and Lawrence Classification

This staging system is used for the evaluation of radiological images of osteoarthritis (OA) [10]:

Grade 0: No features of OA; None
Grade I: Dubious osteophyte, regular joint space; Doubtful
Grade II: Significant osteophyte, possible joint space narrowing; Minimal
Grade III: Moderate osteophytes, moderate narrowing of joint space, mild sclerosis; Moderate
Grade IV: Large osteophytes, marked narrowing of the joint space, significant subchondral bone sclerosis in the form of cysts; Severe

#### 2.1.2. Numerical rating pain scale (NRS)

This scale ranged from 0 to 10, with 0 indicating “no pain” and 10 indicating “unbearable pain.” Patients selected the number that best described their level of pain.

#### 2.1.3. Western Ontario and McMaster Universities Arthritis (WOMAC) Index

The WOMAC Index is widely used in the evaluation of hip and knee OA and includes three different dimensions: pain, stiffness, and physical function. It consists of 5 questions for pain intensity, 2 questions for joint stiffness, and 17 questions for physical function. Each question is scored from 0 to 4 (0: “none,” 1: “mild,” 2: “moderate,” 3: “severe,” 4: “very severe”). Total scores range from 0 to 100, with lower scores indicating satisfactory disease status and higher scores indicating poor disease status [11,12].

For statistical analysis, IBM SPSS Statistics 25 (IBM Corp., Armonk, NY, USA) was used. The Shapiro–Wilk test was employed for normality tests of continuous variables. The sample analyzed in the study was considered dependent. Since normality was not observed in the variables, the Friedman signed rank test was used for comparisons with more than two levels, the Wilcoxon signed rank test was used for comparisons of two groups, and the Mann–Whitney test was used for independent comparisons of two groups. Due to the lack of normality, correlations between continuous variables were analyzed using Spearman rho correlation coefficients. The significance level was set at 0.05 for all tests performed. Sample size and power calculations determined that the inclusion of 41 patients would provide sufficient power (power of 0.92, α = 0.05) using G*Power 3.1 [13,14].

## 3. Results

A total of 41 patients, including 34 women and 7 men aged between 55 and 74 years, were included in the study. The mean age of the patients was 64.95 ± 5.23 years. The mean duration of complaints was 5.66 ± 2.63 years. WOMAC scores and NRS values were obtained for all patients before the injection and at 1, 3, 6, 9, and 12 months after the injection. Regression in synovial hypertrophic tissue, as evaluated via ultrasound before the injection, was observed after the injection. Regarding the obtained p-values, statistically significant differences were found across all groups for WOMAC scores (p < 0.05). The mean WOMAC score before the injection was significantly higher than the mean at all other time points. The mean WOMAC score 1 month after the injection was notably lower than the mean values at other time points, and the mean WOMAC score increased progressively over time as measured at 3, 6, 9, and 12 months after the injection, respectively. The smallest difference in mean scores was found between the 6-month and 9-month marks. Additionally, the differences in standard deviation sizes were noteworthy. There was less change in WOMAC scores before the injection and 12 months after the injection compared to the mean values, with greater variability between patients at 3, 6, 9, and 12 months (Table 1).

In Table 2, the outcomes of the Wilcoxon signed test are presented for comparisons of mean NRS scores and standard deviations before the injection and after injection in the 3rd, 6th, 9th, and 12th months. NRS scores were calculated before the injection and were found to be significantly higher than the mean values at other times. While there was no difference between the mean NRS scores of the 1st and 3rd months after the injection or between the 3rd and 9th months, there were significant differences upon comparing the other mean values (p < 0.05). Correspondingly, it was found that the mean NRS score increased over time, similarly to the WOMAC score.

## 4. Discussion

Following this treatment method applied for patients with knee OA accompanied by synovial hypertrophy, we observed an increase in functional capacity, a decrease in pain scores, and regression in hypertrophic tissue as seen in follow-up ultrasound imaging.

OA is widely recognized as a common cause of knee pain in middle-aged to elderly individuals. Treatment protocols have often involved intraarticular injection techniques and current advancements in treatment methods are progressing rapidly [15,16]. However, studies assessing synovial tissue hypertrophy are still limited in the literature. Synovial changes have been identified at different stages of OA development, emphasizing the importance of the synovium in the pathogenesis of OA [17]. In this context, inflammation is notably prominent. Felson et al. evaluated the severity of inflammation using magnetic resonance imaging and suggested that synovitis should be considered an independent risk factor for OA [18]. Tissue hypertrophy develops as this process continues. The use of intraregional steroids in the treatment of hypertrophic scar tissue, which is a common condition in dermatology and plastic surgery practice, has been ongoing for many years. This scar tissue forms due to a fibroproliferative disorder where inflammation plays a key role. Therefore, intraregional steroids could be used to leverage their antiinflammatory effects in treatment [19]. Given the similarities in the formation mechanisms, the effect of intraregional steroid use on synovial tissue hypertrophy in OA must be considered [19]. Synovial thickening, accompanied by inflammation or localized proliferation, is observed in approximately 50% of patients with knee OA. The impact of intraarticular steroid injections on synovial tissue has been demonstrated in various studies [20,21].

In a study by Neill et al., synovial tissue volume was monitored using magnetic resonance imaging following intraarticular steroid injections in the knee. Among 120 patients evaluated, a decrease in synovial tissue volume was observed during the 6-month follow-up period [21]. Similarly, Keen et al., in a study on hand OA, reported both symptomatic relief and regression of synovial inflammation as evaluated by ultrasound following intramuscular steroid treatment [22].

In our study, we observed both symptomatic relief and a significant decrease in synovial hypertrophic tissue volume. Altman et al. highlighted the statistically significant effect of reducing pain in both the short term (≤3 months) and long term (6–12 months) in their metaanalysis evaluating intraarticular saline administration for the treatment of knee OA. Although the physiological mechanism behind this effect is not yet fully understood, it has been suggested that it may be related to the removal of inflammatory mediators, which cause pain, and the extraction of proteoglycans and aggrecans from the superficial cartilage matrix component [23]. We suggest that joint lavage with a high volume of 0.9% NaCl solution after aspiration of the existing effusion may have a positive effect on pain alleviation.

This study has certain limitations, including its retrospective nature and the absence of a control group. Furthermore, cartilage thickness was not evaluated, despite some studies suggesting that intraarticular steroid therapy may accelerate the degenerative process of cartilage. Consequently, there is a need for randomized controlled prospective studies that include larger patient groups and utilize various imaging methods, such as magnetic resonance imaging, and follow-up parameters, such as the examination of surrounding muscle tissue and predisposing factors, for a more comprehensive analysis.

In conclusion, this study has demonstrated the significant effect of steroid injection into hypertrophic tissue, which can be an option in the treatment of synovitis and subsequent synovial hypertrophy, considered an independent risk factor in the development of OA, on both pain reduction and improvement in functional capacity. We believe that our findings on the effectiveness of this treatment method may offer guidance for future studies.

## Figures and Tables

**Figure:**
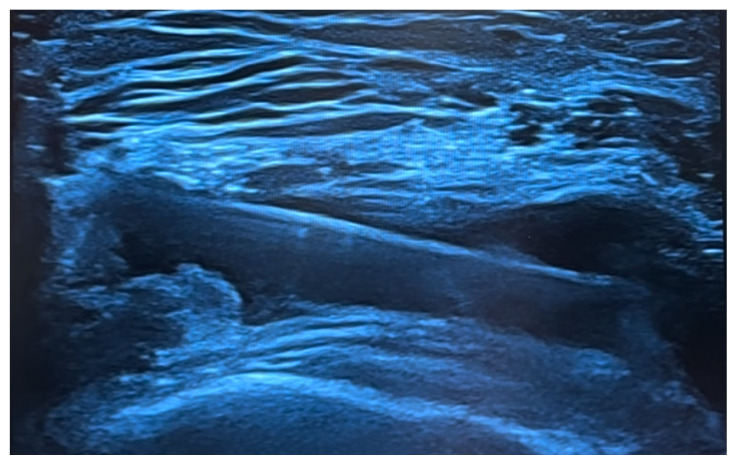
Knee intraarticular injection approach.

**Table 1:** Differences in patients’ mean WOMAC scores and standard deviations.

	WOMAC score					
	Before injection (73.54 ± 7.42)	1st month after injection (4.73 ± 6.10)	3rd month after injection (7.88 ± 9.65)	6th month after injection (13.29 ± 13.62)	9th month after injection (14.00 ± 13.23)	12th month after injection (22.46 ± 12.75)
Before injection (73.54 ± 17.42)	-	p = 0.000	p = 0.000	p = 0.000	p = 0.000	p = 0.001
1st month after injection (4.73 ± 6.10)		-	p = 0.016	p = 0.000	p = 0.000	p = 0.000
3rd month after injection (7.88 ± 9.65)			-	p = 0.000	p = 0.000	p = 0.000
6th month after injection (13.29 ± 13.62)				-	p = 0.038	p = 0.000
9th month after injection (14.00 ± 13.23)					-	p = 0.000
12th month after injection (22.46 ± 12.75)						-

WOMAC: Western Ontario and McMaster Universities Arthritis Index; p < 0.05.

**Table 2:** Differences in patients’ mean NRS scores and standard deviations.

	NRS					
	Before injection (7.78 ± 1.51)	1st month after injection (0.59 ± 0.84)	3rd month after injection (0.90 ± 1.30)	6th month after injection (1.39 ± 1.63)	9th month after injection (1.05 ± 1.32)	12th month after injection (2.54 ± 1.69)
Before injection (7.78 ± 1.51)	-	p = 0.000	p = 0.000	p = 0.000	p = 0.000	p = 0.001
1st month after injection (0.59 ± 0.84)		-	p = 0.060	p = 0.001	p = 0.011	p = 0.000
3rd month after injection (0.90 ± 1.30)			-	p = 0.009	p = 0.364	p = 0.000
6th month after injection (1.39 ± 1.63)				-	p = 0.040	p = 0.000
9th month after injection (1.05 ± 1.32)					-	p = 0.000
12th month after injection (2.54 ± 1.69)						-

NRS: Numerical Rating Pain Scale; p < 0.05.
